# Carbohydrate digestive enzyme inhibition, hepatoprotective, antioxidant and antidiabetic benefits of *Persea americana*

**DOI:** 10.1038/s41598-022-26801-y

**Published:** 2023-01-06

**Authors:** Christian Omonzokpea Ehikioya, Aishat Mary Osagie, Sylvia Oghogho Omage, Kingsley Omage, Marshall Arebojie Azeke

**Affiliations:** 1 Department of Biochemistry, Faculty of Life Sciences, Ambrose Alli University, Ekpoma, Edo State Nigeria; 2 Department of Biochemistry, Faculty of Life Sciences, University of Benin, Benin City, Edo State Nigeria; 3 Department of Biochemistry, College of Basic Medical Sciences, Igbinedion University Okada, Okada, Edo State Nigeria; 4 Department of Human Nutrition, College of Medical, Veterinary and Life Sciences, University of Glasgow, Glasgow, UK; 5 Department of Nutritional Biochemistry and Physiology, Institute of Nutrition, Friedrich Schiller University Jena, Jena, Germany; 6 Division of Endocrinology, Diabetology and Nephrology, Department of Internal Medicine, University Hospital Tübingen, Tübingen, Germany

**Keywords:** Biochemistry, Physiology, Diseases

## Abstract

The medicinal use of *Persea americana* in the treatment of some diseases like hypertension, diabetes, is often with dearth of supporting scientific proof. Thus, we evaluated its ethnomedicinal benefits for possible scientific justification. Thirty healthy Wistar rats were randomly grouped in fives. Alloxan was used to induce diabetes in the rats in groups II to VI. The diabetic rats in group II were treated with glibenclamide, while those in group III were not treated. Also, the diabetic rats in groups IV to VI were treated with the ethanol extracts of the stem bark, leaf, and root of *P. americana* respectively. The parts of *P. americana* comparatively possess highest amounts of phenols (250.50 ± 0.68—bark), saponin (436.80 ± 3.76—leaf), flavonoid (382.80 ± 0.67—leaf) and tannins (58.34 ± 0.09—root). The extracts exhibited high reducing property (FRAP and total reducing), as well as high ABTS and DPPH free radical scavenging ability. The enzyme (alpha-glycosidase and alpha-amylase) inhibitory activity of *P. americana* increases with increasing concentration of the extracts. Administration of methanol extracts of *P. americana* bark, leaf and root to alloxan-induced diabetic rats resulted in significant (P < 0.05) decreases in AST, ALP, ALT, Total bilirubin, LPO, plasma glucose and significant (P < 0.05) increases in GSH, CAT and SOD. These effects were like that of glibenclamide. The enzyme inhibitory, hepatoprotective, antioxidant and antidiabetic properties of *P. americana* are some of the benefits derived from its consumption and ethnomedicinal use.

## Introduction

An estimation by the World Health Organisation indicated that about 80% of the world’s population still practise the use of medicinal herbs as part of their primary healthcare^1^. This practice has been in use, for a long time, in the treatment of different diseases especially among local populations in developing countries^2–6^. However, the practice of traditional indigenous medicine is often associated with reports of successful treatment of diseases with medicinal plants, with no tangible scientific backing^7^. For example, the fruit of the common medicinal plant, *P. americana*, which is often eaten as food, has been reported to be useful in the treatment of hypertension, diabetes, stomach ache, bronchitis etc.^8,9^. Different parts of *P. americana* have been reportedly used for the treatment of different ailments. For example, the stem bark is used in the treatment of asthma, cough, diabetes, fever, as well as diseases of the liver and kidney, and the leaf is used in the treatment of hypertension, cough, ulcer and diarrhoea, while the root is used in the treatment of skin blemishes, convulsion, epilepsy and hormonal disorders in women^8,9^.

*Persea americana* (Avocado or Tree’s fruit) is a flowering medicinal plant and a member of the family Lauraceae^10^, which is widely cultivated in the tropical and subtropical regions^11^. It originated from Mexico and Central or South America but was first cultivated in Mexico as early as 500 bc^12^. One of the common indigenous therapeutic uses of *P. americana* is in the management of diabetes, which is on the increase in most part of the world^13^. Diabetes is a chronic disorder of the endocrine system which is associated with other cardiovascular risk factors like hypertension, dyslipidaemia and other microvascular disorders involving the eyes, kidneys and peripheral nerves^14,15^. It commonly occurs due to abnormality in the metabolism of essential nutrients which results from inadequacy and inefficiency of insulin, and often associated with hyperglycaemia and glycosuria^16^.

The resort to herbal remedy, like the use of *P. americana*, in the management of diabetes is mainly due to its easy accessibility, perceived little or no side effects, as well as little or no associated cost^17^. Several research findings have shown that the presence of phytochemicals and phytonutrients, with their associated medicinal values, play major role in the efficacy of medicinal plants^18–22^. These phytochemicals and phytonutrients are believed to be abundant in *P. americana*^17^. Consequently, the consumption of *P. americana* as food or its use as an herbal remedy for the management of diabetes is reported to be due to the medicinal benefits of its phytonutrients and phytochemicals^17^. In an attempt to give a possible scientific validation to these, we investigated the benefits of the consumption of *P. americana* by evaluating: its ability to inhibit some enzyme of carbohydrate metabolism; its hepatoprotective, antioxidant and antidiabetic properties. The antioxidant activity was compared to that of vitamin C while the antidiabetic properties was compared to that of glibenclamide (a standard antidiabetic drug).

## Materials and methods

### Preparation of methanolic extract of *P. americana* parts

Samples of the leaves, bark and roots of *P. americana* were freshly collected from a local garden in Ujemen-Ekpoma in Edo State (located in Latitudes 6° 44′ 262′′ N and Longitude 6°05′ 76′′ E, with an elevation of 364 m above sea level), with verbal permission from the keeper. The formal identification of the samples was done by a Botanist at the herbarium section of the department of Botany, Ambrose Alli University. A voucher specimen, with herbarium number AAUE735, was deposited in a publicly available herbarium. All possible contaminants were removed by carefully washing the samples under running tap water. They were allowed to dry in an airy environment without direct exposure to sunlight. The dried plant samples were then chopped into fine bits and afterwards pulverized into fine powdery form. Exactly 450 g each of the powdered samples were macerated in 2100 ml of 99% methanol for 72 h. The mixtures were stirred at intervals on a magnetic stirrer. Afterwards, the extracts were separated from the pellets by filtration of the mixtures, using a muslin cloth. The extracts were subsequently concentrated using a rotary vacuum evaporator (Re-52A, lab science, England), at 37 °C. The concentrated leaves, bark and root extracts were stored in sterilized sample bottles and refrigerated for further use.

The percentage yield of the extracts were determined as follows;

$$    \% {\text{ yield }} = {\text{  }}\frac{{{\text{Weight}}{\mkern 1mu} \; {\text{of}}{\mkern 1mu} \; {\text{dry}}{\mkern 1mu} \; {\text{extract}}}}{{{\text{Weight}}{\mkern 1mu} \; {\text{of}}{\mkern 1mu} \; {\text{sample}}}} \times 100.   $$

### Phytochemical estimation

Total phenol content of the plant extracts was determined as described by Siddhuraju and Becker^23^, and was expressed as mg of gallic acid equivalent (mg GAE/g extract). The method described by Makkar et al.^24^, was used to determine the total saponin content of the extracts and it was expressed as Quinine equivalent (mg QUE/g extract). The total flavonoid content of the extracts was estimated in line with the reported procedure by Juan and Chou^25^. The estimation of the total tannin content of the extracts was in accordance with the method described by Polshettiwar et al.^26^, and was expressed as tannic acid equivalent (mg TAN/g extract).

### In vitro antioxidant activity estimation

The method described by Makkar et al.^24^ was used in the estimation of the DPPH (2,2 diphenyl-1-picrylhydrazine) free radical scavenging ability of the extracts and was calculated using DPPH scavenging effect (% inhibition). The method described by Re et al.^27^ was employed in the estimation of the ABTS (2,2-azino-bis-3-ethylbenzthiazoline-6-sulphonic acid) radical scavenging ability of the extracts. The total reducing capability of the extracts was determined as described by Oyaizu^28^. For ferric reducing antioxidant power (FRAP), the method described by Benzie and Strain^29^ was used.

### Enzyme inhibition tests

Alpha-amylase test was carried out as described by Worthington^30^, while alpha-glycosidase test was performed as described by Apostolidis et al.^31^. In both cases, glibenclamide was used as the positive control and the inhibitory activity of both enzymes was expressed as percentage inhibition. The IC_50_ of the various interactions between the extracts and the enzymes were determined using a dose–response curve.

### Experimental design for animal study

Mature male and female Wister Albino rats, whose body weights were between 180 and 220 g, were procured from the animal house of the department of Biochemistry, Federal University of Technology Akure. The rats were housed in clean and well disinfected cages in the animal house of the department of Biochemistry (Ambrose Alli University, Ekpoma) and allowed to adjust to the new environment for one week. The animal house was maintained on a 12-h light and dark photoperiod, with standard room temperature and humidity every day. All through the duration of the experiment, the rats had free access to clean water and food. The experimental procedure, which involved two phases, lasted for 28 days.

*Phase 1* The doses responsible for acute toxicity (LD_50_) of the extracts, glibenclamide and alloxan were estimated by the modified Lorke’s method^32^. Firstly, increasing doses of the extracts (1000, 2000 and 3000 mg/kg), glibenclamide (5, 10 and 15 mg/kg) and alloxan (100, 110 and 120 mg/kg) were administered to three groups of four rats each. The rats were monitored for 24 h for signs of toxicity and death. Secondly, further increasing doses of the extracts (4000, 5000 and 6000 mg/kg), glibenclamide (20, 25 and 30 mg/kg) and alloxan (130, 140 and 150 mg/kg) were administered to another set of three groups of four rats and were also monitored for 24 h for signs of toxicity and death. After 24 h, we did not record any death or observe any signs of toxicity such as depression, weakness and loss of appetite. The LD_50_ of the extracts were determined using the second set with higher doses.

*Phase 2 *Thirty rats were randomly grouped in fives. The rats in group I were not induced with diabetes or treated with either the extracts or glibenclamide. The served as the general control group. Diabetes was induced in groups II to VI via intraperitoneal administration of 120 mg/kg alloxan monohydrate (dissolved in cold normal saline). After 48 h of induction, the experimental rats with fasting blood glucose level ≥ 200 mg/dl were classified as diabetic. At the confirmation of experimental diabetes in the rats, treatment with glibenclamide or *P. americana* extracts commenced immediately. The rats in group II were treated with glibenclamide, at a dose of 10 mg/kg body weight, and they served as the positive control. The rats in group III were neither treated with glibenclamide nor the extracts, and they served as the negative control. In group IV, the rats were treated with methanol extract of the stem-bark of *P. americana* (MEPAB) at a dose of 500 mg/kg body weight. Group V rats were treated with methanol extract of the leaves of *P. americana* (MEPAL) at a dose of 500 mg/kg body weight. While, the rats in group VI were treated with methanol extract of the root of *P. americana* (MEPAR) at a dose of 500 mg/kg body weight.

The route of administration of glibenclamide and the extracts was oral, with a gavage, and the treatment lasted for 28 days. At the end of the treatment, the experimental rats were allowed to fast overnight, after which they were euthanized. Blood samples were collected by cardiac puncture (with the use of sterilized needles and syringes) into clean plain sample tubes. The serum in each tube was separated from the blood pellets, by centrifugation at 4000 rpm for 10 min, and refrigerated at − 4 °C till use. Also, known weights of some harvested organs (liver, kidney and heart) were homogenized in ice cold phosphate buffer using mortar and pestle. The homogenates obtained were centrifuged for 15 min at 4000 rmp and the supernatants collected and refrigerated at − 4 °C till use. After the study, the carcases of the rats were properly wrapped in a plastic bag and buried underground.

### Liver function tests

Alanine aminotransferase (ALT) and Aspartate aminotransferase (AST) activities were determined as described by Reitman and Frankel^33^. The method by Kochmar and Moss^34^ was used in the determination of Alkaline phosphatase (ALP) Activity. While, the method by Jendrassik and Grof^35^ was used in the determination of Total Bilirubin (TB)**.**

### Antioxidant determinations

Superoxide dismutase (SOD) activity was determined as described by Misra and Fridovich^36^. Reduced glutathione (GSH) activity was determined as described by Beutler et al.^37^. The method described by Cohen et al.^38^ was employed in the determination of Catalase (CAT) activity**.** While, the method of Rice-Evans et al.^39^ was employed in the determination of Lipid peroxidation (LPO) activity.

### Statistical analysis

Data were subjected to analysis of variance (ANOVA) and Tukey–Kramer multiple comparison test was performed using instant statistical package. The difference was considered statistically significant when P < 0.05.

### Ethics approval and consent to participate

The ethical approval for this research was obtained from the Research Ethics Committee of the Department of Biochemistry, Ambrose Alli University, Ekpoma Nigeria. The use of rats for the study was also according to the Ethical Guidelines Involving Whole Animal Testing of the Research Ethics Committee of the Department of Biochemistry, Ambrose Alli University, Ekpoma Nigeria, and the reporting in the manuscript follows the recommendations in the ARRIVE guidelines. Consent to participate is not applicable.

## Results

### *Persea americana* possess high amount of biologically useful phytochemicals

The phytochemical constituents of the methanol extract of *P. americana* (P.A) root, bark and leaves are presented in Table 1. The results show that the root extract had the highest tannin content when compared to the extracts of the bark and that of the leaves. The leaves possess the highest saponin and flavonoid content, while the bark has the highest phenolic content. The high amounts of the selected phytochemicals, with proven biological properties (as evident in literature), points at the ethnomedicinal benefits of *P. americana*.

**Table 1:** Phytochemical constituents of *P*. *americana.*

	Phenols (mg GAE/g)	Saponin (mg QUE/g)	Flavonoids (mg QUE/g)	Tannin (mg TAN/g)
MEPAL	206.50 ± 0.26^a^	436.80 ± 3.76^a^	382.80 ± 0.67^a^	48.76 ± 0.37^a^
MEPAR	246.10 ± 0.67^c^	141.70 ± 1.70^b^	344.30 ± 0.32^c^	58.34 ± 0.09^b^
MEPAB	250.50 ± 0.68^b^	111.80 ± 0.22^c^	287.90 ± 0.60^b^	49.42 ± 0.26^a^

Values represent mean ± standard deviation of replicate readings. Values with different superscripts ^**(a,b,c)**^ along the same column are significantly different from one another (P < 0.05). Where *MEPAL* Methanol Extract of *P. americana* leave, *MEPAR* Methanol Extract of *P. americana* root, *MEPAB* Methanol Extract of *P. americana* stem bark.

### The in vitro antioxidant analysis of *P. americana* indicate its high ferric reducing and free radical scavenging abilities

**Figure 1:**
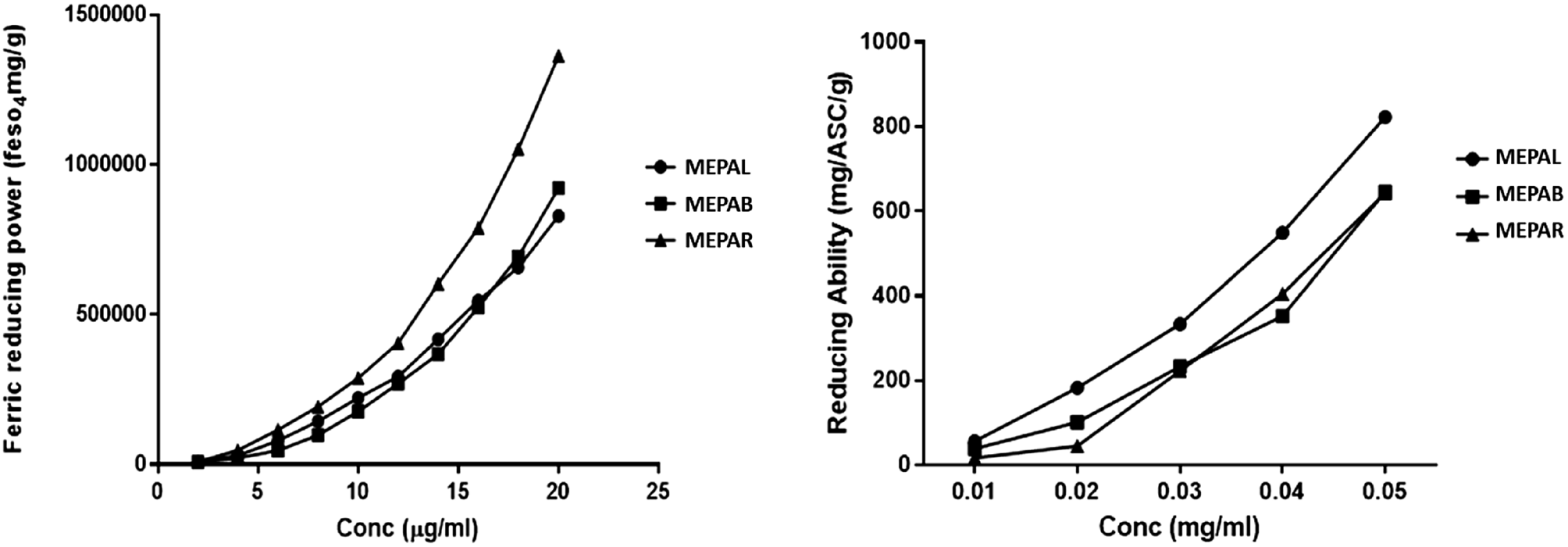
Ferric reducing antioxidant properties (FRAP) and Total reducing activity of *P. americana* extracts. Where *MEPAL* methanol extract of *P. americana* leave, *MEPAR* methanol extract of *P. americana* root, *MEPAB* methanol extract of *P. americana* stem bark.

The ferric reducing antioxidant properties (FRAP) of the extracts as presented in Fig. 1, revealed that the root extract has the highest reducing property compared to the leaf and bark extracts which tend to have similar reducing capacities. The total reducing power of *P. americana* methanol extracts is presented in form of ascorbic acid equivalent (Fig. 1). It indicates that the extract of the leaves has the highest reducing power compared to the root and bark extracts, which tend to have similar reducing capacities. The ABTS radical scavenging ability of the different extracts as presented in Table 2, revealed that the root extract had the highest scavenging ability compare to the bark extract and leaves extract. The DPPH free radical scavenging ability of the different extracts (Table 2) shows that at lower concentrations, the extract of the leaves has the highest scavenging ability, but at the highest concentration tested, the root extract displayed the highest scavenging ability. Also, as indicated in Table 3, the IC_50_ values for the scavenging activities of ABTS and DPPH were highest in the bark extract and lowest in the root extract. The reducing and free radical scavenging activities described above shows that *P. americana* extracts have the potentials to protect against the harmful effects of free radicals in the biological system.

**Table 2:** ABTS and DPPH scavenging activity of *P. americana* extracts.

% Inhibition					
ASSAY	Concentration (µg/ml)	Ascorbic acid	MEPAL	MEPAR	MEPAB
ABTS	40	41.01 ± 0.1^a^	7.86 ± 0.04^b^	7.03 ± 1.51^c^	6.62 ± 0.65^d^
	80	80.25 ± 1.14^a^	12.34 ± 0.45^b^	13.60 ± 0.31^b^	10.12 ± 0.45^c^
	120	90.53 ± 0.45^a^	19.67 ± 0.50^b^	36.98 ± 0.85^c^	13.30 ± 0.14^d^
	160	93.39 ± 0.16^a^	24.05 ± 0.44^b^	43.44 ± 0.44^c^	22.25 ± 0.42^b^
	200	99.04 ± 0.11^a^	28.16 ± 0.49^b^	65.30 ± 0.22^c^	30.58 ± 0.48^b^
DPPH	40	32.78 ± 0.35^a^	0.64 ± 0.13^b^	1.21 ± 0.70^c^	1.95 ± 0.44^d^
	80	48.17 ± 0.24^a^	3.56 ± 1.32^b^	2.63 ± 0.83^c^	2.39 ± 0.73^c^
	120	60.46 ± 0.01^a^	4.39 ± 1.22^b^	3.09 ± 0.66^c^	2.73 ± 0.75^c^
	160	78.04 ± 0.05^a^	8.90 ± 0.82^b^	7.77 ± 0.65^b^	4.94 ± 1.32^c^
	200	90.42 ± 0.12^a^	14.58 ± 1.96^b^	19.34 ± 4.41^c^	11.28 ± 2.19^d^

Values represent mean ± standard deviation of replicate readings. Values with different superscripts ^(a,b,c,d)^ along the same row are significantly different from one another (P < 0.05). Where *MEPAL* methanol extract of *P. americana* leave, *MEPAR* methanol extract of *P. americana* root, *MEPAB* methanol extract of *P. americana* stem bark.

**Table 3:** IC_50_ value for the scavenging activity of ABTS and DPPH by *P. americana* extracts.

IC _50_ (µg/ml)				
ASSAYS	Ascorbic acid	MEPAL	MEPAR	MEPAB
DPPH	102.46 ± 0.12^a^	900.45 ± 18.57^b^	812.50 ± 17.91^c^	1322.76 ± 30.39^d^
ABTS	82.01 ± 0.12^a^	335.86 ± 9.91^b^	170.66 ± 1.25^c^	358 ± 8.42^d^

Values represent mean ± standard deviation of replicate readings. Where *MEPAL* methanol extract of *P. americana* leave, *MEPAR* methanol extract of *P. americana* root, *MEPAB* methanol extract of *P. americana* stem bark. Values with different superscripts ^(a,b,c,d)^ along the same row are significantly different from one another (P < 0.05).

### *Persea americana* effectively inhibits some enzymes (alpha-glycosidase and alpha-amylase) of carbohydrate metabolism in vitro

The inhibitory effect *P. americana* extracts on alpha-glycosidase activity as presented in Table 4 shows that the alpha-glycosidase inhibitory activity of the extracts increased with increasing concentrations of the extract as compared to that of glibenclamide (positive control). *P. americana* root extract had the highest inhibitory activity (95.00 ± 1.20%) followed by the leaf extract (83.68 ± 5.11%) and bark extract (73.77 ± 6.81%). As indicated in Table 4, the alpha amylase inhibitory activity of the extracts also increased with increasing concentrations of the extract as compared to the positive control, glibenclamide. The leaf extract had the highest inhibitory ability (94.80 ± 2.89%) followed by the bark extract (93.26 ± 1.16%) and root extract (90.37 ± 0.96%). The IC_50_ value of the extracts on alpha-amylase and alpha-glycosidase activities were significantly lower than that of glibenclamide (Table 5). However, the root extract had the highest value on alpha-amylase while the bark extract had the highest value on alpha-glycosidase. The inhibitory effects of the extracts on alpha-glycosidase and alpha-amylase shows the potential of *P. americana* to reduce or slow down carbohydrate metabolism, which is useful in the management of diabetes.

**Table 4:** Inhibition of alpha-glycosidase and alpha-amylase activity by extracts of *P. americana.*

%Inhibition					
ASSAY	Concentration (µg/ml)	Glibenclamide	MEPAL	MEPAB	MEPAR
Alpha-glycosidase	0.4	56.07 ± 9.63^a^	56.36 ± 2.40^a^	55.36 ± 0.60^a^	63.46 ± 1.10^b^
	0.8	66.38 ± 0.48^a^	66.77 ± 9.41^a^	61.26 ± 1.90b	73.87 ± 1.50^c^
	1.2	64.35 ± 9.83^a^	72.67 ± 4.91^b^	66.47 ± 5.91^a^	77.58 ± 2.60^c^
	1.6	58.77 ± 14.5^a^	83.68 ± 5.11^b^	73.77 ± 6.81^c^	95.00 ± 1.20^d^
Alpha-amylase	0.4	42.77 ± 4.63^a^	63.97 ± 2.12^b^	73.03 ± 4.24^c^	63.78 ± 0.77^b^
	0.8	41.81 ± 3.85^a^	83.82 ± 3.28^b^	86.03 ± 2.22^b^	73.22 ± 2.87^c^
	1.2	44.41 ± 4.91^a^	86.71 ± 2.12^b^	90.37 ± 2.31^c^	81.41 ± 3.76^d^
	1.6	39.11 ± 0.19^a^	94.80 ± 2.89^b^	93.26 ± 1.16^b^	90.37 ± 0.96^c^

Values represent mean ± standard deviation of replicate readings. Values with different superscripts ^(a,b,c,d)^ along the same row are significantly different from one another (P < 0.05). Where *MEPAL* methanol extract of *P. americana* leave, *MEPAR* methanol extract of *P. americana* root, *MEPAB* methanol extract of *P. americana* stem bark.

**Table 5:** IC_50_ value of *P. americana* extracts on alpha-amylase and alpha-glycosidase activity.

IC_50_ (mg/ml)				
Assays	Glibenclamide	MEPAL	MEPAB	MEPAR
Alpha amylase	1.45 ± 0.10^a^	0.69 ± 0.003^b^	0.68 ± 0.02^b^	0.74 ± 0.01^c^
Alpha glycosidase	0.97 ± 0.03^a^	0.81 ± 0.06^b^	0.90 ± 0.06^c^	0.73 ± 0.01^d^

Values represent mean ± standard deviation of replicate readings. Where *MEPAL* methanol extract of *P. americana* leave, *MEPAR* methanol extract of *P. americana* root, *MEPAB* methanol extract of *P. americana* stem bark. Values with different superscripts ^(a,b,c,d)^ along the same row are significantly different from one another (P < 0.05).

### The acute toxicity test of *P. americana* indicates its relative safety as administered to the experimental diabetic rats

In Table 6, the acute toxicity tests (LD_50_) of the extracts, glibenclamide and alloxan on the experimental animals indicate that there were no signs of mortality or any behavioural changes. However, hypoactivity was observed at the highest dose of alloxan administration. Thus, the extracts were well tolerated by the experimental rats and are relatively safe.

**Table 6:** Acute toxicity (LD_50_) test of *P. americana* leaf, seed and root extract, as well as glibenclamide and alloxan.

Extract/drug	Phase	N	Dose (mg/kg)	Toxicity sign	Mortality
MEPAL	1	4	1000	No sign	Nil
		4	2000	No sign	Nil
		4	3000	No sign	Nil
	2	4	4000	No sign	Nil
		4	5000	No sign	Nil
		4	6000	No sign	Nil
MEPAB	1	4	1000	No sign	Nil
		4	2000	No sign	Nil
		4	3000	No sign	Nil
	2	4	4000	No sign	Nil
		4	5000	No sign	Nil
		4	6000	No sign	Nil
MEPAR	1	4	1000	No sign	Nil
		4	2000	No sign	Nil
		4	3000	No sign	Nil
	2	4	4000	No sign	Nil
		4	5000	No sign	Nil
		4	6000	No sign	Nil
Glibenclamide	1	4	5	No sign	Nil
		4	10	No sign	Nil
		4	15	No sign	Nil
	2	4	20	No sign	Nil
		4	25	No sign	Nil
		4	30	No sign	Nil
Alloxan	1	4	100	No sign	Nil
		4	110	No sign	Nil
		4	120	No sign	Nil
	2	4	130	No sign	Nil
		4	140	Overactivity	Nil
		4	150	Overactivity	Nil

*N* number of rats, *MEPAL* methanol extract of *P. americana* leaf, *MEPAB* methanol extract of *P. americana* bark, *MEPAR* methanol extract of *P. americana* root.

### *Persea americana* restores loss in body weight but had no significant effect on the weight of some selected organs (Liver, Kidney, Heart) of the experimental diabetic rats

Table 7 shows steady significant (P < 0.05) decreases in body weights of the experimental diabetic rats treated with glibenclamide (group II) up to week 3 of treatment. At week 4 of treatment, the body weight significantly increased up to the initial weight. This trend was also observed in the extract treated groups (groups IV, V and VI), with a significant (P < 0.05) increase in body weight at week 4 of treatment. In contrast, the rats in group III which were induced with diabetes but not treated exhibited steady significant (P < 0.05) decreases in body weight up to the last week of the experiment (week 4). Thus, *P. americana* root extracts restore and maintains body weight in the alloxan-induced diabetic rats.

**Table 7:** Effect of *P. americana* extracts on the body weight and selected organs (Liver, Kidney, Heart) of alloxan–induced diabetic rats.

Body/organ	Time		Weight (g) of groups					
			I	II	III	IV	V	VI
Body	Before experiment		183.0 ± 0.78^a^	197.3 ± 5.98^a^	202.8 ± 2.84^a^	200.5 ± 8.65^a^	195.7 ± 5.38^a^	192.2 ± 3.94^a^
	Week 1		184.6 ± 0.41^a^	193.7 ± 6.15^b^	196.9 ± 3.17^b^	193.7 ± 7.16^b^	189.3 ± 4.83^b^	187.6 ± 4.36^b^
	Week 2		186.3 ± 0.92^a^	193.4 ± 5.06^b^	192.7 ± 3.06^c^	190.4 ± 6.99^c^	188.3 ± 5.06^b^	185.8 ± 4.42^c^
	Week 3		188.2 ± 0.84^b^	195.7 ± 5.63^b^	190.0 ± 3.24^d^	191.8 ± 6.88^c^	190.0 ± 4.70^b^	187.1 ± 4.10^b^
	Week 4		191.1 ± 1.07^b^	197.5 ± 6.06^c^	188.6 ± 3.46^d^	194.4 ± 6.87^b^	192.0 ± 4.61^c^	188.6 ± 4.14^b^
Liver	Week 4		5.64 ± 0.50^a^	7.40 ± 0.91^b^	7.66 ± 0.71^b^	6.07 ± 0.14^a^	5.99 ± 0.19^a^	6.65 ± 0.43^a^
Kidney	Week 4		1.00 ± 0.07^a^	1.33 ± 0.05^b^	1.43 ± 0.18^b^	0.15 ± 0.04^a^	1.11 ± 0.07^a^	1.33 ± 0.11^a^
Heart	Week 4		0.56 ± 0.02^a^	0.69 ± 0.02^b^	0.81 ± 0.03^c^	0.62 ± 0.04^a^	0.68 ± 0.04^b^	0.65 ± 0.05^b^

Values represent mean ± standard deviation (n = 5). Values with different superscripts ^(a,b,c,d)^ along the same group are significantly different from one another (P < 0.05). Group I—normal control rats, Group II—diabetic rats treated with glibenclamide, Group III—diabetic rats not treated, Group IV—diabetic rats treated with methanol extract of *P. americana* bark, Group V—diabetic rats treated with methanol extract of *P. americana* leaves, Group VI—diabetic rats treated with methanol extract of *P. americana* root.

As also shown in Table 7, the administration of extracts of *P. americana* stem bark, leaf and root did not significantly affect the weight of the selected organs of the rats in the respective treated groups (IV, V and VI). On the contrary, the untreated (group III) and glibenclamide-treated (group II) diabetic groups showed significant (P < 0.05) increase in organ weight, as compared with the normal control group. Thus, as against the effect of glibenclamide, the extracts of *P. americana* has no toxic effect on the selected organs.

### *Persea americana* protects against the deleterious effects of alloxan on the hepatocytes by preventing the leakage of the liver function enzymes, thereby reducing their levels in the blood

Table 8 shows the effect of *P. americana* extracts on the activities of some liver function enzymes. The activities of the enzymes were shown to be significantly (P < 0.05) lower in the extract-treated diabetic groups as compared to those of the untreated diabetic groups. Also, the effects of the extracts were shown to be similar to that of the standard drug (glibenclamide). There were significant (P < 0.05) decreases in the AST levels in all the extract-treated groups (IV, V and VI) as compared to that of the untreated group (III—negative control). Between the extract-treated diabetic groups, that of the root extract (group VI) was significantly (P < 0.05) lower as compared to other extracts treated groups and the glibenclamide-treated group (II—positive control). Also, the AST level of the glibenclamide-treated group was significantly (P < 0.05) lower than that of the untreated group (III-negative control)". ALP activity was significantly (P < 0.05) lower in the extract-treated groups (IV, V and VI) as compared to that of the untreated group (III), but significantly (P < 0.05) higher than that of the glibenclamide-treated group (II). However, among the extract-treated groups, the leaf and back extracts were shown to be more effective than the root extract. ALT activity was significantly (P < 0.05) lower in the extract-treated groups (IV, V and VI) as compared to that of the untreated group (III) and that of the glibenclamide-treated group (II). However, there were no significant differences among the extract-treated groups. The total bilirubin level, as observed, was also significantly (P < 0.05) lower in the extract-treated groups (IV, V and VI) as compared to that of the untreated group (III), but significantly (P < 0.05) higher than that of the glibenclamide-treated group (II). However, among the extract-treated groups, the leaf extract was shown to be more effective. The reduction of the plasma levels of these enzymes by the extracts indicates their protective roles against the hepatotoxic effects of alloxan in the experimental rats.

**Table 8:** Effect of *P. americana* extracts on the liver enzymes of alloxan-diabetic rats.

Groups	Enzyme assays			
	AST (IU/l)	ALP (IU/l)	ALT (IU/l)	Total Bilirubin (mg/100 ml)
I	11.60 ± 0.93a	6.59 ± 0.59^a^	5.60 ± 0.81^a^	1.36 ± 0.12^a^
II	27.00 ± 1.58^b^	12.51 ± 1.49^b^	17.40 ± 1.21^b^	0.29 ± 0.03^b^
III	57.28 ± 3.44^c^	63.94 ± 1.94^c^	41.32 ± 2.49^c^	2.25 ± 0.13^c^
IV	24.00 ± 2.10^b^	21.24 ± 1.41^d^	13.40 ± 1.17^d^	0.55 ± 0.04^d^
V	29.20 ± 1.35^b^	18.37 ± 1.60^d^	12.00 ± 0.89^d^	0.36 ± 0.03^b^
VI	16.60 ± 1.36^d^	41.25 ± 4.67^e^	13.80 ± 1.24^d^	0.49 ± 0.05^d^

Values represent mean ± standard deviation (n = 5). Values with different superscripts ^(a,b,c,d,e)^ along the same column are significantly different from one another (P < 0.05). Group I—normal control rats, Group II—diabetic rats treated with glibenclamide, Group III—diabetic rats not treated, Group IV—diabetic rats treated with methanol extract of *P. americana* stem bark, Group V—diabetic rats treated with methanol extract of *P. americana* leaves, Group VI—diabetic rats treated with methanol extract of *P. americana* root.

### *Persea americana* extracts protect against oxidative stress by boosting the activities of antioxidant enzymes (GSH, CAT and SOD) and inhibiting lipid peroxidation process in the selected tissues of the experimental diabetic rats.

Table 9 shows the effect of *P. americana* extracts on the GSH activity of the serum, liver, heart and kidney of the experimental rats. GSH activity was significantly (P < 0.05) higher in the blood, liver, heart and kidney of the rats in all the extract-treated diabetic groups (IV, V and VI) as compared to the untreated diabetic group (III). However, among the extract-treated groups, the GSH activity of the bark extract was significantly (P < 0.05) highest in the blood, while that of the root extract was significantly (P < 0.05) highest in the liver and kidney. Comparatively, the administration of glibenclamide (group II) resulted in significant (P < 0.05) increase in GSH activity in the blood and significant (P < 0.05) decrease in GSH activity in the liver.

**Table 9:** Effect of *P. americana* extracts on GSH, CAT, SOD and LPO activities of the alloxan-diabetic rats.

Tissue/organs					
Enzyme activity	Group	Serum	Liver	Heart	Kidney
GSH (mg/g protein)	I	11.59 ± 0.99^a^	9.55 ± 1.17a	4.56 ± 0.41^a^	6.98 ± 0.65^a^
	II	20.36 ± 1.41^b^	9.73 ± 1.57^a^	5.62 ± 0.37^a^	6.59 ± 1.01^a^
	III	8.49 ± 0.37^c^	7.42 ± 0.29^b^	2.86 ± 0.25^b^	4.66 ± 0.33^b^
	IV	14.84 ± 0.99^d^	10.33 ± 1.00^c^	5.09 ± 0.47^c^	7.85 ± 0.95^c^
	V	11.15 ± 1.64^a^	11.25 ± 1.05^c^	4.70 ± 0.46^c^	6.02 ± 0.69^a^
	VI	11.49 ± 1.12^a^	13.62 ± 1.39^d^	5.43 ± 0.37^c^	9.26 ± 0.62^d^
Catalase (nmol/min/ml)	I	0.042 ± 0.001^a^	0.054 ± 0.001^a^	0.054 ± 0.001^a^	0.054 ± 0.001^a^
	II	0.046 ± 0.002^b^	0.056 ± 0.001^a^	0.055 ± 0.003^a^	0.052 ± 0.001^a^
	III	0.036 ± 0.001^c^	0.044 ± 0.001^b^	0.051 ± 0.001^b^	0.052 ± 0.001^a^
	IV	0.044 ± 0.001^b^	0.066 ± 0.003^c^	0.055 ± 0.004^a^	0.059 ± 0.001^b^
	V	0.050 ± 0.001^d^	0.052 ± 0.001^a^	0.053 ± 0.004^a^	0.057 ± 0.001^b^
	VI	0.045 ± 0.001^b^	0.046 ± 0.002^b^	0.055 ± 0.004^a^	0.060 ± 0.001^b^
SOD (U/g protein)	I	1.29 ± 0.10^a^	1.56 ± 0.05^a^	0.92 ± 0.15^a^	1.68 ± 0.02^a^
	II	1.43 ± 0.14^b^	1.59 ± 0.03^a^	1.16 ± 0.04^b^	1.82 ± 0.04^b^
	III	1.48 ± 0.06^b^	1.58 ± 0.02^a^	1.18 ± 0.03^b^	1.79 ± 0.02^b^
	IV	1.75 ± 0.03^c^	1.63 ± 0.03^b^	1.29 ± 0.07^c^	1.88 ± 0.01^c^
	V	1.56 ± 0.03^d^	1.66 ± 0.02^b^	1.31 ± 0.06^c^	1.85 ± 0.01^c^
	VI	1.66 ± 0.01^c^	1.75 ± 0.02^c^	1.26 ± 0.04^c^	1.87 ± 0.03^c^
LPO (U/g protein)	I	9.14 ± 0.66^a^	23.36 ± 2.93^a^	31.58 ± 6.45^a^	91.43 ± 8.13^a^
	II	9.32 ± 1.12^a^	43.84 ± 4.72^b^	45.70 ± 5.55^b^	268.52 ± 14.63^b^
	III	39.94 ± 1.97^b^	95.7 ± 2.05^c^	81.50 ± 3.48^c^	299.64 ± 15.75^c^
	IV	10.29 ± 0.56^a^	26.84 ± 5.69^a^	64.70 ± 4.42^d^	98.64 ± 12.08^d^
	V	7.12 ± 0.65^c^	40.76 ± 7.55^b^	40.13 ± 4.35^e^	272.0 ± 19.05^b^
	VI	26.62 ± 1.23^d^	28.18 ± 7.25^d^	48.77 ± 5.81^b^	247.3 ± 18.75^e^

Values represent mean ± standard deviation (n = 5). Values with different superscripts ^(a,b,c,d,e)^ along the same column are significantly different from one another (P < 0.05). Group I—normal control rats, Group II—diabetic rats treated with glibenclamide, Group III—diabetic rats not treated, Group IV—diabetic rats treated with methanol extract of *P. americana* stem bark, Group V—diabetic rats treated with methanol extract of *P. americana* leaves, Group VI—diabetic rats treated with methanol extract of *P. americana* root.

Also, Table 9 shows the effect of *P. americana* on the CAT activity of the heart, kidney, liver and blood of the experimental rats. Administration of the extracts to the alloxan-induced diabetic rats resulted in significant (P < 0.05) increases in the CAT activity in the blood, liver, heart and kidney of the treated groups (IV, V and VI), as compared to the untreated group (III). In a similar manner, the administration of glibenclamide also resulted in significant (P < 0.05) increases in the CAT activity in all the tissues, except the kidney. Among the extract treated groups, the CAT activity of the leaf extract was significantly (P < 0.05) highest in the blood while that of the bark extract was significantly (P < 0.05) highest in the liver.

In Table 9, the administration of *P. americana* bark, leaf or root extracts resulted in significant (P < 0.05) increases in the SOD activity of the serum, liver, heart and kidney of the alloxan-induced diabetic rats (groups IV, V and VI), as compared to the untreated diabetic group (III) and the glibenclamide-treated diabetic group (II). Within the extract-treated groups, the effect of the bark extract is evidently highest in the serum while that of the root extract is highest in the liver. Interestingly, the effect of the extracts on the SOD activity of the tissues were observed to be better than that of the standard drug, glibenclamide.

Also in Table 9, the administration of the methanol extracts of the different parts of *P. americana* to the alloxan-induced diabetic rats resulted in significant (P < 0.05) decreases in the products of lipid peroxidation (LPO) in the serum, liver, heart and kidney of the treated groups (IV, V and VI), as compared to the untreated diabetic group (III). Administration of glibenclamide also resulted in similar effect in the treated groups, except in the kidney where the reduction was not significant. However, among the extract-treated diabetic groups, the effect of the leaf extract was significantly (P < 0.05) highest in the serum and heart. While the effect of the bark extract was significantly (P < 0.05) highest in the liver and kidney. Evidently, treatment with *P. americana* extracts protects against the actions of free radicals by slowing down the lipid peroxidation process.

### *Persea americana* exhibits antidiabetic properties by reducing the plasma glucose level in alloxan-induced experimental diabetic rats

Table 10 shows the effects of the administration of the bark, leaf and root extracts of *P. americana* in alloxan-induced diabetic rats. The significant (P < 0.05) increases in the glucose level of the diabetic groups, after induction with alloxan, were steadily reversed in the course of treatment (from week 1 to week 4) with the various extracts and glibenclamide. Thus, administration of the extracts resulted in steady significant (P < 0.05) decreases in the plasma glucose level in all the treated groups (IV, V and VI), as compared to that of the untreated group (III). The effects of the extracts are similar to that of the standard antidiabetic drug, glibenclamide.

**Table 10:** Effect of *P. americana* extracts on the blood glucose level of the alloxan-diabetic rats.

Blood glucose level (mg/dl)/time						
Group	Before induction	After induction	Week 1	Week 2	Week 3	Week 4
I	96.0 ± 4.4	97.0 ± 3.8^a^	97.0 ± 2.7^a^	98.0 ± 3.5^a^	95.3 ± 2.9a	95.7 ± 1.9^a^
II	98.3 ± 2.6	596.3 ± 1.8^b^	519.0 ± 6.3^b^	426.7 ± 22.0^b^	201.0 ± 20.3^b^	99.0 ± 2.1^a^
III	98.3 ± 1.8	594.0 ± 2.1^b^	559.3 ± 6.7^c^	489.3 ± 7.7^c^	374.0 ± 12.8^c^	278.0 ± 15.7^b^
IV	97.3 ± 4.1	593.3 ± 2.7^b^	525.0 ± 11.0^b^	390.0 ± 15.3^d^	205.0 ± 11.2^b^	101.3 ± 4.4^a^
V	102.0 ± 2.7	596.0 ± 2.3^b^	546.7 ± 10.7^d^	457.7 ± 25.0^e^	203.7 ± 10.8^b^	99.0 ± 2.7^a^
VI	97.0 ± 1.5	592.7 ± 3.3^b^	533.3 ± 13.3^d^	380.3 ± 13.1^d^	202.0 ± 12.7^b^	105.7 ± 3.0^a^

Values represent mean ± standard deviation (n = 5). Values with different superscripts ^(a,b,c,d,e)^ along the same column are significantly different from one another (P < 0.05). Group I—normal control rats, Group II—diabetic rats treated with Glibenclamide, Group III—diabetic rats not treated, Group IV—diabetic rats treated with methanol extract of *P. americana* bark, Group V—diabetic rats treated with methanol extract of *P. americana* root, Group VI—diabetic rats treated with methanol extract of *P. americana* leaf.

## Discussion

The consumption of medicinal herbs or plants for various health and nutritional benefits has stood the test of time. It is an ancient tradition which is still very much relevant in the modern society, especially in the management of diabetes. In ethnomedicine, the use of *P. americana* to manage or possibly treat diabetes, and its complications, has been well accepted due to the belief that it offers health benefits that are perceived not to be available in orthodox medicine and related therapeutic interventions. In corroboration with the above, findings form our present study show that consumption of *P. americana* comes with some health benefits which are especially useful in the management of diabetes and the complications associated with it. Our quantitative phytochemical screening of *P. americana* shows that the different parts (bark, leaf and root) of the plant possess high amount of biologically useful active principles. Phenols were found to be comparatively higher in the bark extract, saponin and flavonoids were found to be high in the leaf extract, while tannin was found to be abundant in the root extract. These phytochemicals are known for their biological activities and their presence in *P. americana* makes it a plant of high medicinal value. For example, phenols have been reported to exhibit hypoglycemic properties possibly by inhibiting the action of sodium-glucose transporter I^40^. Saponin has also been reported to exhibit hypoglycemic properties by possibly inhibiting glycogenesis or glycolysis in the liver^41^. Flavonoids have been shown to possess powerful antioxidant abilities which is helpful in protecting biological tissues against the activities of toxic agents or oxidative stress^42^. Like phenols, tannins are also reputed for their high hypoglycemic activities^40^. Thus, the biological properties of the selected phytochemicals, as described above points at the ethnomedicinal benefits of *P. americana.*

In vitro antioxidant analysis of the bark, leaf and root extracts of *P. americana* shows high reducing (FRAP) and free radical scavenging (ABTS and DPPH) activities which are equivalent to that of ascorbic acid. Antioxidants play a major role in the body’s defense system against reactive oxygen species (ROS) ^43^. These antioxidant properties, which could be linked to its high flavonoid content, indicate the ability of *P. americana* to protect against the harmful effects of free radicals in the biological system. This agrees with earlier report that the seeds and leaves of *P. americana* can be useful in the formulation of future antioxidant products^44^, which can be attributed to the presence of phytochemicals like flavonoids in the leaf as described above. Apart from the antioxidant properties, the in vitro analysis also revealed the effectiveness of *P. americana* at inhibiting the activities of some enzymes of carbohydrate metabolism. The effect was observed to be similar to that of the standard antidiabetic drug (glibenclamide). Alpha-glycosidase and alpha-amylase are major enzymes that are involved in the catabolism of carbohydrates. Their inhibition often plays a major role in the regulation of blood glucose level by reducing the amount of glucose available from breakdown of carbohydrates. In line with our findings, other researchers have earlier reported the ability of *P. americana* to reduce glycemic indices in vivo^41,44^. The inhibitory effects of the extracts of the different parts of *P. americana* on alpha-glycosidase and alpha-amylase shows its potential to reduce or slow down carbohydrate metabolism, which is very useful in the management of diabetes and its resulting complications. These effects could also be attributed to the presence of high amounts of saponin and phenols in the plant, as observed in our phytochemical screening.

Acute toxicity test (LD_50_) of the bark, leaf and root extracts of *P. americana* indicates its relative safety in vivo. After administering the different extracts to the normal experimental rats, no signs of mortality or behavioural changes were observed, even at the highest dose of 6000 mg/kg body weight. Acute toxicity test is often used to quickly assess the relative safety of natural products or medicinal plants in the biological system^2^. Thus, our observation shows that the extracts were well tolerated by the experimental rats, which is indicative of the relative safety of the use of *P. americana* in ethnomedicine. This is also supported by the finding that, administration of the extracts to alloxan-induced diabetic rats did not cause any changes in the weights of the selected organs (liver, kidney and heart), rather they tend to normalise them. This effect was observed to be similar to that of glibenclamide. However, the administration of the extracts to alloxan-induced diabetic rats was helpful in restoring and maintaining the body weights of the experimental rats, after the effect of alloxan. Alloxan-induced weight loss in the untreated diabetic rats imitates the commonly observed clinical signs and symptoms of diabetics^45^. The ability of *P. americana* to restore body weight may be connected to its hypoglycaemic effect, possibly due to increased glucose metabolism, or the prevention of muscle wasting, as a protective role.

The protective role of *P. americana* was also observed in the liver of the alloxan-induced diabetic rats. Alloxan evidently caused increased activities of AST, ALP, ALT and total bilirubin in the serum of the alloxan-induced diabetic rats due to its toxic effect on the hepatocytes. The membranes of the hepatocytes were possibly destroyed by the activities of free radicals, as initiated by alloxan toxicity. Destruction of the membranes of the hepatocytes often results in the escape of resident enzymes (AST, ALP, ALT) from the hepatocytes to into the blood. Thus, membrane permeability of hepatocytes increases during liver injury which leads to leakage of hepatic enzymes from hepatocytes. Increased levels of ALP and bilirubin are indicative of biliary tract obstruction while elevated levels of ALT and AST are known markers of liver injury. High amount of these enzymes in the blood is often of diagnostic value because it is indicative of damage to the liver. However, treatment with the bark, leaf and root extracts of *P. americana* was found to protect against the deleterious effects of alloxan on the hepatocytes. Administration of the extracts to the experimental diabetic rats resulted in the reduction of the activities of these enzymes in the blood, by preventing their escape from the hepatocyte. The effects of the extracts were shown to be similar to that glibenclamide. The reduction of the plasma levels of these enzymes and total bilirubin by the extracts indicates their protective roles against the hepatotoxic effects of alloxan in the experimental rats. This may be connected with the activities of phytochemicals like flavonoids which are reportedly abundant in the plant.

Oxidative stress is a known mechanism by which free radicals cause damage in the biological system. The toxicity of alloxan is often mediated through the actions of free radicals. The reduction of the activities or destruction of these free radicals by antioxidants usually protects biological molecules, membranes and tissues from damage. High amount of these antioxidant enzymes in the biological system is therefore useful and necessary for the fight against the damage by free radicals. Our findings show that the administration of *P. americana* extracts to the alloxan-induced diabetic rats protects the selected tissues (blood, liver, heart and kidney) against oxidative stress or damage caused by alloxan. As compared to the untreated diabetic groups, treatment with the extracts resulted in increased activities of the antioxidant enzymes (GSH, CAT and SOD) and decreased amount of product of lipid peroxidation in the selected tissues of the alloxan-induced diabetic rats. These effects were similar to that of glibenclamide. However, it is worthy of note that the effect of the extracts on the SOD activity of the tissues were observed to be better than that of glibenclamide. Evidently, treatment with *P. americana* extracts protects against the actions of free radicals by boosting the activities of GSH, CAT and SOD, as well as slowing down the destructive process of lipid peroxidation. These effects are a function of the high reducing and free radical scavenging potential of *P. americana*, as well as the presence of high amounts of relevant phytochemicals, like flavonoids.

The antidiabetic property of *P. americana* was tested in alloxan-induced diabetic rats. Administration of alloxan caused increases in plasma glucose level of the experimental rats, by destruction of the pancreatic cells through free radical mechanism. The comparative increased plasma glucose level in the untreated-diabetic group was maintained up to the fourth week of the experiment. However, treatments with the extracts of *P. americana* were shown to be effective as there were steady decreases in the plasma glucose level in all the extract-treated diabetic groups, from week 1 to week 4. The reductions in plasma glucose occasioned by the actions of the extracts are similar to that by glibenclamide. The antidiabetic property of *P. americana* can be attributed, partly, to its ability to inhibit the actions of alpha-glycosidase and alpha-amylase as described above and as previously reported^41,46–48^.

## Conclusion

Our study shows that *P. americana* possess high amount of biologically useful phytochemicals. In vitro antioxidant analysis indicates its high ferric reducing and free radical scavenging abilities, as well as its ability to effectively inhibit the activities of alpha-glycosidase and alpha-amylase. Acute toxicity test indicates the relative safety of *P*. *americana*. The bark, leaf and root extracts of *P*. *americana* exhibit hepatoprotective, antioxidant and antidiabetic properties. Thus, the use of *P*. *americana* can serve as basis for the development of good alternatives to available synthetic drugs for glycaemic management. However, the limitation of this study is that its findings are still not sufficient enough for clinical applications of the plant.

## Data Availability

The datasets used and/or analysed during the current study available from the corresponding author on reasonable request.

## References

[1] World Health Organization. WHO traditional Medicine Strategy 2002–2005. http://www.who.int/medicine/Library/trm/trm-strat-eng.pdf.

[2] Omage K, Azeke AM, Orhue NEJ, Iseghohi OS (2017). Toxicological implications of the therapeutic use of *Acalypha wilkesiana* leaves in tradition medicine. Clin. Phytosci..

[3] Omage K, Azeke AM, Omage OS (2018). Evaluation of the efficacy of *Acalypha wilkesiana* leaves in managing cardiovascular disease risk factors in rabbits exposed to salt-loaded diets. Clin. Phytosci..

[4] Omage OS, Orhue NEJ, Omage K (2019). Evaluation of the phytochemical content, in vitro antioxidant capacity, biochemical and histological effects of *Dennettia tripetala* fruits in healthy rats. Food Sci. Nutr..

[5] Nwangwu CO, Josiah JS, Usunobun U, Nwangwu U, Akintola AA, Ehiremen OI, Omage K (2011). Effects of aqueous and ethanolic extract of *Vernonia amygdalina* leaf on the plasma lipid profile and liver function parameters of normal rats. Curr. Res. J. Biol. Sci..

[6] Josiah JS, Nwangwu CO, Omage K, Abdulrahamon AA, Nkwonta B, Aderoju OF (2012). Possible revival of atrophied islet cells of the pancreas by *Vernonia amygdalina* in alloxan induced diabetic rats. J. Appl. Pharm. Sci..

[7] Chindo AB, Anuka AJ, Gamaniel SK (2012). Toxicity screenings of *Ficus platyphylla* stem bark in rats. Pharmacologia..

[8] Muanya CA, Odukoya OA (2008). Lipid peroxidation as index of activity in aphrodisiac herbs. J. Plant Sci..

[9] Oluwole FS, Onasanwo SA, Olaleye SB (2011). Effects of aqueous and methanolic extracts of Persea Americana Leaf (Avocado Pear) on gastric acid secretion in male albino rats. Eur. J. Sci. Res..

[10] Morton JF (1987). Avocado: Fruits of Warm Climates.

[11] Lu QY, Arteaga JR, Zhang Q, Huerta S, Go VL, Heber D (2005). Inhibition of prostate cancer cell growth by an avocado extract: Role of lipid-soluble bioactive substances. J. Nutr. Biochem..

[12] Duester KC (2000). Avocados a look beyond basic nutrition for one of nature's whole foods. Nutr. Today.

[13] Kumar R, Ilavarasan T, Jayachandran M, Deecaraman P, Aravindan N, Padmanabhan L, Krishan MRV (2008). Anti-diabetic activity of Syzygiumcumini and its isolated compound against streptozotocin-induced diabetic rats. J. Med. Plants Res..

[14] Grundy MS, Benjamin JI, Burke LG, Chait AE, Howard VB, Mitch M (1999). Diabetes and cardiovascular disease: A statement for health care professionals from the American Heart Association. Circulation.

[15] Barnett, H. A. & O’Gara, G. *Diabetes and the Heart. Clinical Practice Series*. 7–30 (Churchill Livingstone, 2003).

[16] Chatterjea, M. N. & Rana, S. Diabetes mellitus. In *Textbook of Medical Biochemistry*, 5th Ed. (ed. Jaypee, B.) 315–320 (Medical Publishers Ltd, 2002).

[17] Jarald E, Joshi SB, Jain DC (2008). Diabetes and herbal medicines. Iran. J. Pharmacol. Ther..

[18] Ganesan K, Xu B (2018). A critical review on phytochemical profile and health promoting effects of mung bean (*Vigna radiata*). Food Sci. Hum. Wellness.

[19] Omage K, Azeke AM (2014). Medicinal potential of *Acalypha wilkesiana* leaves. Adv. Res..

[20] Unhunmwangho SE, Omage K, Erifeta OG, Josiah JS, Nwangwu COS (2013). Possible reversal of sodium arsenate-induced liver toxicity by hexane leaf extract of *Alchornea laxiflora*. Asian J. Med. Sci..

[21] Omage AAM, Orhue NEJ (2015). Implications of oral administration of extracts of *Acalypha wilkesiana* leave on serum electrolytes, urea and creatinine in normal experimental rabbits. Biokemistri.

[22] Omage K, Onoagbe OI, Erifeta OG, Uhunmwangho SE, Ajeigbe OK, Amegor OF (2011). Effects of aqueous root extract of *Treculia africana* on blood glucose, lipid profile and body weight changes of Streptozotocin-induced diabetic and normal rats. Int. J. Plant Physiol. Biochem..

[23] Siddhuraju P, Becker K (2007). The antioxidant and free radical scavenging activities of processed cowpea (*Vigna unguiculata*). seed extracts. J. Food Chem..

[24] Makkar HP, Siddhuraju P, Becker K (2007). Methods in Molecular Biology: Plant Secondary Metabolites.

[25] Juan MT, Chou CC (2010). Enhancement of antioxidant activity, total phenolic and flavonoid content of black Soybeans by solid state fermentation with *Bacillus subtilis* BCRC 14715. Food Microbiol..

[26] Polshettiwar SA, Ganjiwale RO, Wadher SJ, Yeole PG (2007). Spectrophotometric estimation of total tannins in some ayurvedic eye drops. Indian J. Pharm. Sci..

[27] Re R, Pellegrini N, Proteggente A, Pannala A, Yang M, Rice-Evans C (1999). Antioxidant activity applying an improved ABTS radical cation decolourization assay. Free Rad. Biol. Med..

[28] Oyaizu M (1986). Studies on products of browning reactions: Antioxidative activities of products of browning reaction prepared from glucosamine. Jpn. J. Nutr..

[29] Benzie IFF, Strain JJ (1999). Ferric reducing antioxidant power assay: Direct measure of total antioxidant activity of biological fluids and modified version for simultaneous measurement of total antioxidant power and ascorbic acid concentration. Methods Enzymol..

[30] Worthington, V. Alpha amylase. In *Worthington Enzyme Manual*. 36–41 (1993).

[31] Apostolidis E, Kwon Y, Shetty K (2007). Inhibitory potential of herb, fruit, and fungal-enriched cheese against key enzymes linked to type 2diabetes and hypertension. Innov. Food Sci. Emerg. Technol..

[32] Lorke D (1983). A new approach to practical acute toxicity testing. Arch. Toxicol..

[33] Reitman S, Frankel S (1957). Glutamic pyruvate transaminase assay by colorimetric method. Am. J. Clin. Pathol..

[34] Kochmar JF, Moss DW (1976). Fundamentals of Clinical Chemistry.

[35] Jendrassik L, Grof P (1938). Colorimetric method of determination of bilirubin. Biochemistry.

[36] Misra HP, Fridovich I (1972). The role of superoxide anion in the autoxidation of epinephrine and a simple assay for superoxide dismutase. J. Biol. Chem..

[37] Beutler E, Duron O, Kelly BM (1963). Improved method for the determination of blood glutathione. J. Lab. Clin. Med..

[38] Cohen G, Dembiec D, Marcus J (1970). Measurement of catalase activity in tissue extracts. Annu. Rev. Biochem..

[39] Rice-Evans C, Omorphos CS, Baysal E (1986). Sickle cell membrane and oxidative damage. Biochem. J..

[40] Huang S, Czech MP (2007). The GLUT4 glucose transporter. Cell Metab..

[41] Oboh G, Ademosun AO, Akinleye M, Omojokun OS, Boligon AA, Athayde ML (2015). Starch composition, glycemic indices, phenolic constituents, and antioxidative and antidiabetic properties of some common tropical fruits. J. Ethnol. Foods..

[42] Huyut, Z., Beydemir, Ş., Gülçin, İ. *Antioxidant and Antiradical Properties of Selected Flavonoids and Phenolic Compounds*. 1–10 (Biochemistry Research International, 2017). 10.1155/2017/7616791 PMC5660747 29158919

[43] Vivek KG, Surendra KS (2006). Plants as natural antioxidants. Nat. Prod. Radia..

[44] Alhassan A, Sule M, Lawal A (2017). In vitro inhibitory activities of *Persea americana* seed extracts on α-amylase and α-glucosidase. Bayero J. Pure Appl. Sci..

[45] World Health Organization. *Definition, Diagnosis and Classification of Diabetes Mellitus and its Complications. Part 1: Diagnosis and Classification of Diabetes Mellitus (WHO/NCD/NCS/99.2)* (1999).

[46] Ajani A, Olanrewaju BO (2014). Avocado pear fruits and leaves aqueous extracts inhibit α-amylase, α-glucosidase and snap induced lipid peroxidation—An insight into mechanisms involve in management of type 2 diabetes. Int. J. Appl. Nat. Sci..

[47] Uysal S, Zengin G, Aktumsek A, Karatas S (2015). Fatty acid composition, total sugar content and anti-diabetic activity of methanol and water extracts of nine different fruit tree leaves collected from Mediterranean region of turkey. Int. J. Food Prop..

[48] Pahua-Ramos ME, Garduño-Siciliano L, Dorantes-Alvarez L (2014). Reduced-calorie avocado paste attenuates metabolic factors associated with a hypercholesterolemic-high fructose diet in rats. Plant Foods Hum. Nutr..

